# Supplementary material for: Evolutionarily distant I domains can functionally replace the essential ligand-binding domain of Plasmodium TRAP
Source: eLife. 2020 Jul 10;9:e57572. doi: 10.7554/eLife.57572 (PMC7351488; doi:10.7554/eLife.57572)
Supplement: Supplementary file 1. — Primers used for the generation and genotyping of the parasite lines presented in this study. [file elife-57572-supp1.docx]

**Supplementary File 1. Primer sequences.**

Primers used for the generation and genotyping of the parasite lines presented in this study.

**Number Sequence (5’ to 3’)**

**P134** GAGCATACAAAAATACATGCACAC

**P137** TGATTTACTTCCATCATTTTGCCC

**P165** CCCAAGCTTTGCCTTTAAATAATAAACTCATAAACTCG

**P166** GGGGTACCctccaaacaaaaaatggacacg

**P171** GAATACATGTAAAAAAGAGAAATTCCTTCG

**P174** GTAAAATAAGCGATATAGAAGGGAGC

**P234** CTTGCACCGGTTTTTATAAAATTTTTATTTATTTATAAGC

**P508** ATCCCGCGGTACATGTGCATATAATAAAATTTGTTGGTTGTAATAA

TTAGC

**P509** TAGGATATCCTCCAAACAAAAAATGGACACGTGCAACTA

**P516** CCTAGCTAGCttacttgtacagctcgtccatg

**P535** CCCTTATAAAAAGACATATGAAGCTCTTAGGAAATAG

**P536** CTATTTCCTAAGAGCTTCATATGTCTTTTTATAAGGG

**P537** AATGACTGGAACTAATTTTAATTAACATATATATC

**P538** GATATATATGTTAATTAAAATTAGTTCCAGTCATT

**P549** AAGAGCAACTTTTTCTACTTCCTGACAAACTTTAG

**P600** CCCAAGCTTCAAAAAAGCAGGCTTGCCGC

**P601** GCCGATATCCAAGAAAGCTGGGTGGTACCC

**P691** GCGGCAAGCCTGCTTTTTTGAAG

**P700** CAGACGGAGATTCCGGCAGGTTG

**P788** GGCCTGCAGCCCAGCTTAATTC

**P951** TCACCTTCAGCTTGGCG

**P1199** GAAAGTATTTGTCAGCAGTAACATGTGC

**P1200** GATTGCTACTGCGGCGAAATTC

**P1201** GACCATCACTGGTATTCGTGCTG

**P1202** GTCAAGTTCGTGGTGCCGTG

**P1340** tgaggccggtgctgagtatgtcg

**P1341** ccacagtcttctgggtggcagtg

**P1344** aagcattaaataaagcgaatacatccttac

**P1345** ggagattggttttgacgtttatgtg

**P1550** GGTCAAGTTCGTGGTGCCTTC
