# Supplementary material for: Evolutionarily distant I domains can functionally replace the essential ligand-binding domain of Plasmodium TRAP
Source: eLife. 2020 Jul 10;9:e57572. doi: 10.7554/eLife.57572 (PMC7351488; doi:10.7554/eLife.57572)
Supplement: Supplementary file 2. — Shown are the sequences of each TRAP replacement. Residues that are part of the extendable ß-ribbon are written in green, residues that form the remainder of the I domain are written in red, residues of the thrombospondin domain are written in orange, and the remaining native residues of PbTRAP are written in black. Residues written in blue were introduced into wild type PbTRAP to generate a more negative charge on the portion of the I domain surface surrounding the MIDAS in the RevCharge mutant. Residues written in white on a black background were mutated to create a better fitting of the exchanged portion of the I domain with the N- and C-terminal segments of the PbTRAP I domain/extendable ß-ribbon. The calculated pI of the I domain region is shown in parentheses. [file elife-57572-supp2.docx]

**Supplementary File 2. Amino acid sequences of the TRAP variants expressed by the parasite lines *TRAP-I*, *MIC2-I*, *αX-I,* *αL-I, and RevCharge*.**

Shown are the sequences of each TRAP replacement. Residues that are part of the extendable ß-ribbon are written in green, residues that form the remainder of the I domain are written in red, residues of the thrombospondin domain are written in orange, and the remaining native residues of *Pb*TRAP are written in black. Residues written in blue were introduced into wild type *Pb*TRAP to generate a more negative charge on the portion of the I domain surface surrounding the MIDAS in the *RevCharge* mutant. Residues written in white on a black background were mutated to create a better fitting of the exchanged portion of the I domain with the N- and C-terminal segments of the *Pb*TRAP I domain/extendable ß-ribbon. The calculated pI of the I domain region is shown in parentheses.

***Pb*TRAP-αL**

MKLLGNSKYFFVVLLLCISVFLNGQEILDEIKYSEEVCNEQSDLVFLFDGSMSLQPDEFQKILDFMKDVMKKLSNTSYQFAAVQFSTSYKTEFDFSDYVKRKDPDALLKHVKHMLLLTNTFGAINYVATEVFREELGARPDATKVLIIITDGEATDSGNIDAAKDIIRYIIGIGKHFQTKESQETLHKFASKPASEFVKILDTFEKLKDLFTELQKKICQEVEKVALCGKWEEWSECSTTCDNGTKIRKRKVLHPNCAGEMTAPCKVRDCPPKPVAPPVIPIKVPDVPVKPVEPIEPAEPAEPAEPAEPAEPAEPAEPAEPAEPAEPAEPAEPAEPAEPAEPAEPAEPAEPAEPAKPAEPAEPAEPAEPAEPVNPDNPILPIKPEEPSGGAEPLNPEVENPFIIPDEPIEPIIAPGAVPDKPIIPEESNELPNNLPESPSDSQVEYPRPNDNGDNSNNTINSNKNIPNKHVPPTDDNPYKGQEERIPKPHRSNDEYIYYNNANNNDKLEPEIPSKDYEENKSKKQSKSNNGYKIAGGIIGGLAIIGCIGVGYNFIAGSSAAAMAGEAAPFEDVMADDEKGIVENEQFKLPEDNDWN (pI 5.8)

***Pb*TRAP-αX**

MKLLGNSKYFFVVLLLCISVFLNGQEILDEIKYSEEVCNEQQDIVFLIDGSGSISSRNFATMMNFVRAVISQFQRPSTQFSLMQFSNKFQTHFTFEEFRRSSNPLSLLASVHQLQGFTYTATAIQNVVHRLFHASYGARRDAAKILIVITDGKKEGDSLDYKDVIPMADAAGIIRYAIGVGLAFQNRNSWKELNDIASKPSQEHIFKVEDFDALKDIQNQLKEKICQEVEKVALCGKWEEWSECSTTCDNGTKIRKRKVLHPNCAGEMTAPCKVRDCPPKPVAPPVIPIKVPDVPVKPVEPIEPAEPAEPAEPAEPAEPAEPAEPAEPAEPAEPAEPAEPAEPAEPAEPAEPAEPAEPAEPAKPAEPAEPAEPAEPAEPVNPDNPILPIKPEEPSGGAEPLNPEVENPFIIPDEPIEPIIAPGAVPDKPIIPEESNELPNNLPESPSDSQVEYPRPNDNGDNSNNTINSNKNIPNKHVPPTDDNPYKGQEERIPKPHRSNDEYIYYNNANNNDKLEPEIPSKDYEENKSKKQSKSNNGYKIAGGIIGGLAIIGCIGVGYNFIAGSSAAAMAGEAAPFEDVMADDEKGIVENEQFKLPEDNDWN (pI 8.9)

***Pb*TRAP-MIC2**

MKLLGNSKYFFVVLLLCISVFLNGQEILDEIKYSEEVCNEQVDICFLIDSSGSIGIQNFRLVKQFLHTFLMVLPIGPEEVNNAVVTYSTDVHLQWDLQSPNAVDKQLAAHAVLEMPYKKGSTNTSDGLKACKQILFTGSRPGREHVPKLVIGMTDGESDSDFRTVRAAKEIRELGGIVTVLAVGHYVKHSECRSMCGCSGTSDDDSPCPLYLRADWGQLATAIKPMLKEVCQEVEKVALCGKWEEWSECSTTCDNGTKIRKRKVLHPNCAGEMTAPCKVRDCPPKPVAPPVIPIKVPDVPVKPVEPIEPAEPAEPAEPAEPAEPAEPAEPAEPAEPAEPAEPAEPAEPAEPAEPAEPAEPAEPAEPAKPAEPAEPAEPAEPAEPVNPDNPILPIKPEEPSGGAEPLNPEVENPFIIPDEPIEPIIAPGAVPDKPIIPEESNELPNNLPESPSDSQVEYPRPNDNGDNSNNTINSNKNIPNKHVPPTDDNPYKGQEERIPKPHRSNDEYIYYNNANNNDKLEPEIPSKDYEENKSKKQSKSNNGYKIAGGIIGGLAIIGCIGVGYNFIAGSSAAAMAGEAAPFEDVMADDEKGIVENEQFKLPEDNDWN (pI 6.1)

***Pb*TRAP**

MKLLGNSKYFFVVLLLCISVFLNGQEILDEIKYSEEVCNEQIDLHILLDGSGSIGHSNWISHVIPMLTTLVDNLNISRDEINISMTLFSTYARELVRLKRYGSTSKASLRFIIAQLQNNYSPHGTTNLTSALLNVDNLIQKKMNRPNAIQLVIILTDGIPNNLKKSTTVVNQLKKKDVNVAIIGVGAGVNNMFNRILVGCGKLGPCPYYSYGSWDQAQTMIKPFLSKVCQEVEKVALCGKWEEWSECSTTCDNGTKIRKRKVLHPNCAGEMTAPCKVRDCPPKPVAPPVIPIKVPDVPVKPVEPIEPAEPAEPAEPAEPAEPAEPAEPAEPAEPAEPAEPAEPAEPAEPAEPAEPAEPAEPAEPAKPAEPAEPAEPAEPAEPVNPDNPILPIKPEEPSGGAEPLNPEVENPFIIPDEPIEPIIAPGAVPDKPIIPEESNELPNNLPESPSDSQVEYPRPNDNGDNSNNTINSNKNIPNKHVPPTDDNPYKGQEERIPKPHRSNDEYIYYNNANNNDKLEPEIPSKDYEENKSKKQSKSNNGYKIAGGIIGGLAIIGCIGVGYNFIAGSSAAAMAGEAAPFEDVMADDEKGIVENEQFKLPEDNDWN (pI 9.7)

***Pb*RevCharge**

MKLLGNSKYFFVVLLLCISVFLNGQEILDEIKYSEEVCNEQIDLHILLDGSGSIGESNWISEVIPMLTTLVDNLNISRDEINISMTLFSTYARELVRLKRYGSTSKASLRFIIAQLQNNYSPEGTTNLTSALLNVDNLIQKKMNRPNAIQLVIILTDGIPNNLQDSTTVVNQLKKKDVNVAIIGVGAGVNNMFNEILVGCGALGPCPYYSYGSWDQAQTMIKPFLSKVCQEVEKVALCGKWEEWSECSTTCDNGTKIRKRKVLHPNCAGEMTAPCKVRDCPPKPVAPPVIPIKVPDVPVKPVEPIEPAEPAEPAEPAEPAEPAEPAEPAEPAEPAEPAEPAEPAEPAEPAEPAEPAEPAEPAEPAKPAEPAEPAEPAEPAEPVNPDNPILPIKPEEPSGGAEPLNPEVENPFIIPDEPIEPIIAPGAVPDKPIIPEESNELPNNLPESPSDSQVEYPRPNDNGDNSNNTINSNKNIPNKHVPPTDDNPYKGQEERIPKPHRSNDEYIYYNNANNNDKLEPEIPSKDYEENKSKKQSKSNNGYKIAGGIIGGLAIIGCIGVGYNFIAGSSAAAMAGEAAPFEDVMADDEKGIVENEQFKLPEDNDWN (pI 6.8)
