# Supplementary material for: Evolutionarily distant I domains can functionally replace the essential ligand-binding domain of Plasmodium TRAP
Source: eLife. 2020 Jul 10;9:e57572. doi: 10.7554/eLife.57572 (PMC7351488; doi:10.7554/eLife.57572)
Supplement: Transparent reporting form [file elife-57572-transrepform.pdf]

eLIFE
1st Floor
24 Hills Road
Cambridge CB2 1JP, UK
P 01223 855340
W elifesciences.org
T @elife

eLife's transparent reporting form

We encourage authors to provide detailed information *within their submission* to facilitate the interpretation and replication of experiments. Authors can upload supporting documentation to indicate the use of appropriate reporting guidelines for health-related research (see EQUATOR Network), life science research (see the BioSharing Information Resource), or the ARRIVE guidelines for reporting work involving animal research. Where applicable, authors should refer to any relevant reporting standards documents in this form.

If you have any questions, please consult our Journal Policies and/or contact us: editorial@elifesciences.org.

Sample-size estimation

- You should state whether an appropriate sample size was computed when the study was being designed
- You should state the statistical method of sample size computation and any required assumptions
- If no explicit power analysis was used, you should describe how you decided what sample (replicate) size (number) to use

Please outline where this information can be found within the submission (e.g., sections or figure legends), or explain why this information doesn't apply to your submission:

For each parasite line at least three different batches of mosquitoes were infected at different dates and with different donor mice. Only exception to this rule were the non-fluorescent parasite lines *TRAP-I*, *MIC2-I*, *aX-I* and *aL-I* that were only fed once because these lines served only as additional controls to their respective fluorescent counterparts (*TRAP-I fluo*, *MIC2-I fluo*, *aX-I fluo* and *aL-I fluo*). The number of technical replicates was dependent on the performed experiment. Important experiments like sporozoite countings within a specific tissue (midgut, hemolymph or salivary glands) were usually performed twice or more per mosquito batch while *in vivo* experiments were performed two to three times with 2-4 mice per experiment. The exact number of biological and technical replicates of a given graph is stated in the respective figure legend.
A minimum of three independent feeding experiments per parasite line is, state of our knowledge, standard in the field. Given the complexity of the *Plasmodium* life cycle and dependent on the number of parasite lines involved this is a reasonable number of replicates that can be done in a manageable time frame.

Replicates

- You should report how often each experiment was performed
- You should include a definition of biological versus technical replication
- The data obtained should be provided and sufficient information should be provided to indicate the number of independent biological and/or technical replicates
- If you encountered any outliers, you should describe how these were handled
- Criteria for exclusion/inclusion of data should be clearly stated
- High-throughput sequence data should be uploaded before submission, with a private link for reviewers provided (these are available from both GEO and ArrayExpress)

Please outline where this information can be found within the submission (e.g., sections or figure legends), or explain why this information doesn't apply to your submission:

The number of repeats for each experiment is stated in the legend of the respective figure. We also clearly differentiated between technical and biological replicates. Biological replicates are defined as the number of mosquito batches that were independently infected (with different donor mice at different dates). Technical replicates are defined as the number of experiments performed with the same batch of mosquitoes.
We didn't encounter outliers and didn't exclude any data.
This study contains no high-throughput sequencing data.

Statistical reporting

- Statistical analysis methods should be described and justified
- Raw data should be presented in figures whenever informative to do so (typically when N per group is less than 10)
- For each experiment, you should identify the statistical tests used, exact values of N, definitions of center, methods of multiple test correction, and dispersion and precision measures (e.g., mean, median, SD, SEM, confidence intervals; and, for the major substantive results, a measure of effect size (e.g., Pearson's r, Cohen's d)
- Report exact p-values wherever possible alongside the summary statistics and 95% confidence intervals. These should be reported for all key questions and not only when the p-value is less than 0.05.

Please outline where this information can be found within the submission (e.g., sections or figure legends), or explain why this information doesn't apply to your submission:

Statistical analysis methods were choosen based on the number of compared conditions and the distribution of data within each condition. For data sets with <3 conditions students t test was used to identify statistical significant differences while for data sets >2 the one-way-ANOVA test was used. The respective test was adjusted according to the distribution of data in a gaussian or non-gaussian manner.
The used statistical analysis methods are stated in the figure legends as well as definitions of center, precision measures and p-values. The exact values of N are either stated in the figure legend (e.g. for *in vivo* experiments) or indicated within graphs (e.g. above each column/condition).

(For large datasets, or papers with a very large number of statistical tests, you may upload a single table file with tests, Ns, etc., with reference to sections in the manuscript.)

Group allocation

- Indicate how samples were allocated into experimental groups (in the case of clinical studies, please specify allocation to treatment method); if randomization was used, please also state if restricted randomization was applied
- Indicate if masking was used during group allocation, data collection and/or data analysis

Please outline where this information can be found within the submission (e.g., sections or figure legends), or explain why this information doesn't apply to your submission:

We didn't make use of clinical samples or included data generated in clinical studies. Therefore we didn't use methods for sample allocation.
To safeguard good scientific practice all parasite lines were analyzed independently by two to three different researchers.

Additional data files ("source data")

- We encourage you to upload relevant additional data files, such as numerical data that are represented as a graph in a figure, or as a summary table
- Where provided, these should be in the most useful format, and they can be uploaded as "Source data" files linked to a main figure or table
- Include model definition files including the full list of parameters used
- Include code used for data analysis (e.g., R, MatLab)
- Avoid stating that data files are "available upon request"

Please indicate the figures or tables for which source data files have been provided:

With few exceptions data presented in figures are also given as nummerical data in table format within the manuscript. Therefore we didn't provide any additional source data.
